# The highest region of muscle spindle abundance should be the optimal target of botulinum toxin A injection to block muscle spasms in rats

**DOI:** 10.3389/fneur.2023.1061849

**Published:** 2023-02-23

**Authors:** Jie Yu, Yunshan Li, Lu Yang, Yi Li, Shibin Zhang, Shengbo Yang

**Affiliations:** ^1^Department of Anatomy, Zunyi Medical University, Zunyi, China; ^2^Department of Clinical Medicine, Zunyi Medical University, Zunyi, China; ^3^Laboratory Animal Center, Zunyi Medical University, Zunyi, China

**Keywords:** muscle spasm, muscle spindle, gastrocnemius muscle, botulinum toxin A, neuromuscular blockade

## Abstract

**Purpose:**

The effective relief of muscle spasticity requires prompt solutions in rehabilitation medicine. This study aimed to reveal that the highest region of muscle spindle abundance is the optimal target of botulinum toxin A (BTX-A) injections for relieve muscle spasm.

**Methods:**

Sixty adult, male Sprague–Dawley rats with lower limbs spasm caused by stroke after modeling, weighing (200 ± 20) g, were included in this study. The modelrats were divided into four groups: muscle spasm model group (group A), model rats treated with BTX-A injections into the middle of the muscle belly (group B), model rats treated with BTX-A injections into the center of the intramuscular nerve-dense region (INDR) (group C), and model rats treated with BTX-A injection into the center of the highest region of muscle spindle abundance (HRMSA) (group D). Groups B, C, and D were further divided into two subgroups: the 3rd and 6th days after BTX-A injection. The rats in each group were assigned modified Ashworth scale scores (MAS), and the changes in gastrocnemius muscle tone, wet muscle weight, and cross-sectional area of muscle fiber were detected.

**Results:**

Muscle spindle abundance was the highest in the upper part of the INDR. Group B experienced no significant changes in MAS, muscle tone, wet muscle weight, or cross-sectional area of the muscle fiber. Conversely, groups C and D experienced a decrease in these indicators. Group C experienced the most significant decrease in wet muscle weight and cross-sectional area of muscle fibers. Group D experienced the most notable decrease in MAS and muscle tone. There were no significant differences in the indicators between the 3rd and 6th days after BTX-A injections in group B and there were significant differences in the improvement in the indicators between the two subgroups in groups C and D, with group D experiencing more notable intersubgroup differences.

**Conclusion:**

The efficacy of BTX-A injections into the HRMSA is significantly superior to that of conventional BTX-A injections into the middle of the belly muscle or the INDR in the treatment of muscle spasms. Hence, HRMSA should be the optimal target of BTX-A to relieve muscle spasms.

## Introduction

Muscle spasticity usually occurs secondary to many central nervous system diseases, such as stroke, brain trauma, spinal cord injury, and lateral sclerosis (1). Muscle spasticity limits patient movement and affects their daily activities. Hence, the effective relief of muscle spasticity remains an important issue that requires a prompt solution in rehabilitation medicine.

At present, the intramuscular injection of botulinum toxin A (BTX-A) into motor endplates to block acetylcholine release at the presynaptic membrane serves as a common treatment option for muscle spasticity (2). However, many problems may arise during the administration of injections because the staining and localization of motor endplates in human muscles are limited by the availability of fresh specimens (3). In clinical practice, the middle part (the most bulky) of the muscle belly is often selected as the target of BTX-A injections because motor endplates are located in the middle of the muscle fibers (4, 5). However, the position of the motor endplate band may vary depending on the anatomical structure of each muscle, thus possibly resulting in the decreased efficacy of BTX-A injections into the thickest of the muscle belly (6, 7).

Recent studies have shown that the location of intramuscular nerve-dense regions (INDRs) coincides with that of the motor endplate bands. INDRs may serve as an alternative target of BTX-A blocking injections for muscle spasticity (6–8). Therefore, INDR localization has become a research focus (9, 10). Some studies revealed that the excitation of muscle spindles could exacerbate muscle spasticity *via* the α-γ loop (11) and that muscle spindle abundance is high in INDRs but are not evenly distributed (12).

In this study, we selected the highest region of muscle spindle abundance (HRMSA) in the INDR as the new target of BTX-A injections for treating muscle spasticity. This study aimed to compare the efficacy of this therapeutic approach with that of conventional BTX-A injection into the middle of the muscle belly and to prove that HRMSA is the optimal target of BTX-A injections for muscle spasticity.

## Materials and methods

### Experimental animals and ethics

Sixty adult, male Sprague–Dawley (SD) rats (SPF grade) weighing (200 ± 20) g obtained from the Laboratory Animal Center of Zunyi Medical University (Production No.SCXK-2021-0002; Use No. SYXK-2021-0004) were included in this study. Among the included 60 rats, 6 rats were used for Sihler's staining of intramuscular nerves, 6 were used to determine the HRMSA in the INDR by haematoxylin–eosin (HE) staining, and 6 were used to localize the center of the INDR (CINDR) and the center of the HRMSA (CHRMSA) with spiral computed tomography (CT). The remaining 42 rats were used to create animal models of muscle spasticity secondary to stroke. Based on previous studies, limb spasms were most obvious from the 3rd day to the 9th day after the establishment of the rat stroke model (13), and there was obvious efficacy on the 3rd day after BTX- A injection (14). the model rats were divided into four groups: the muscle spasm model group (group A, six rats), model rats treated with conventional BTX-A injections into the middle of the muscle belly (group B, 12 rats), model rats treated with BTX-A injections into the CINDR (group C, 12 rats), model rats treated with BTX-A injections into the CHRMSA (group D, 12 rats). Groups B, C, and D were further divided into two subgroups: the 3rd day after BTX-A injection and the 6th day after BTX-A injection (i.e., group B1/B2, group C1/C2, and group D1/D2). All experimental procedures were performed under the approval of the Animal Experiment Ethics Committee of Zunyi Medical University (No: 2021-2-026).

### Sihler's staining of intramuscular nerves

The rats were anesthetized by spontaneous sevoflurane inhalation and were sacrificed by decapitation. The entire triceps of the lower leg were removed, fixed in 10% formalin for 1 month, and macerated with 3% potassium hydroxide solution containing 0.2% hydrogen peroxide for 3 weeks. The macerated muscles were subsequently decalcified in Sihler's solution I (one part of glacial acetic acid, two parts of glycerine, and 12 parts of 1% aqueous chloral hydrate) for 3 weeks, stained with Sihler's solution II (one part of Ehrlich's haematoxylin, two parts of glycerine, and 12 parts of 1% aqueous chloral hydrate) for 4 weeks, destained in Sihler's solution I for 3–24 h, neutralized in 0.05% lithium carbonate solution for 2 h, and then hyalinised using glycerine gradients [40, 60, 80, and 100% (1 week in each concentration)]. The location of the intramuscular nerve-branch-dense region was observed under an X-ray view box and then photographed. The relative location of the INDR and its center along the muscle belly length in percentile measures was determined using a micrometer.

### HE staining of muscle spindles and localization of HRMSA

The INDR in the medial head of the gastrocnemius (MG) and the lateral head of the gastrocnemius (LG) in six rats were divided into upper, middle, and lower parts of equal length. The muscles were weighed, fixed in formalin, dehydrated, immersed, and embedded in paraffin wax. The paraffin-embedded tissue samples were then continuously sectioned, stained using HE, and observed under a microscope (OLYMPUS, Japan) before the muscle spindles were reconstructed. The predicted number of muscle spindles was calculated using the following formula: S_pn_ = 20.5${\text{m}}_{\text{n}}^{0.49}$, where S_pn_ is the predicted number of muscle spindles, and m_n_ is the muscle weight (12). The muscle spindle abundance was obtained by dividing the actual number of muscle spindles by the predicted number of muscle spindles. The muscle spindle abundance was compared among the different parts of the INDR. The intramuscular location of CHRMSA in the INDR was determined on the basis of the results of Sihler's staining.

### Creation of an animal model-of muscle spasticity secondary to stroke

The rats were anesthetized with intraperitoneal injections of 3% pentobarbital sodium (35 mg/kg). A longitudinal incision was made slightly lateral to the neck midline to separate the common carotid artery and the vagus nerve by using blunt dissection. Proximal end ligation of the common carotid artery and external carotid artery was performed, and the internal carotid artery was clipped using a microvascular clamp. After an incision was made at a distance of 5 mm from the common carotid artery bifurcation, the intraluminal thread (0.26 mm in diameter; Beijing Getimes Technology Co., Ltd., China) was advanced 18–20 mm into the internal carotid artery through the incision until mild resistance was felt. Subsequently, the arterial clamp was released, and the proximal end of the internal carotid artery was ligated together with the intraluminal thread. Finally, the wound was rinsed and sutured before intraperitoneal injections of penicillin were administered to prevent infections. On the 3rd day after creating the rat model, the rats' limb movements were observed, and the intracranial infarction were visualized using magnetic resonance imaging (MRI) (General Electric, US). The neurological impairment was assessed using Zea Longa scores. Modified Ashworth scale (MAS) outcomes were assessed before and after each intervention by a blinded rater who is the use of well-trained, experienced testers. The MAS was used to quantify the extent of spasticity and each test movement was performed for 1 second before determining spasticity. Data from model groups showed ankle MAS. For data analysis, the 0 value of the MAS was assigned as 1; 1 was assigned as 2; 1+ was assigned as 3 and so on (15). The BL-420 biological signal acquisition system (Chengdu Techman Software Co., Ltd., China) was used to detect the changes in muscle tone.

### Localization of CINDR and CHRMSA by spiral CT

The curve line close to the skin connecting the lateral femoral epicondyle (point a) and the medial femoral epicondyle (point b) posterior to the knee joint was considered as the horizontal reference line (H), and the curve line connecting the lateral femoral epicondyle and the lateral malleolus (point c) was considered as the longitudinal reference line (L). One silk thread soaked in barium sulfate evenly mixed with glue was sutured on the designed H and L reference lines. The CINDR and CHRMSA were marked by barium sulfate that was evenly mixed with glue. The points marked by the barium sulfate were punctured with a syringe needle perpendicular to the coronal plane. Thereafter, 16-row spiral CT scanning (General Electric, US; slice thickness: 0.625 mm; helical pitch: 0.562:1; automatic tube current in milliamperes: 210 mA; voltage: 140 kV) and 3D reconstruction were performed. The GEAW4.5 postprocessing operating system (General Electric, US) was used to measure the length of the H and L reference lines. The intersection of the vertical line passing through the body surface's puncture point P (P_1_, P_2_, P_3_, and P_4_ represent the body surface projection points of CINDR and CHRMSA in LGand MG, respectively) with the H reference line and the horizontal line with the L reference line were recorded as P_H_ (P_1H_ to P_4H_) and P_L_ (P_1L_ to P_4L_), respectively. The length of the curve line connecting point a and P_H_ was H', and the length of the curve line connecting point a and P_L_ was L'. The H'/H × 100% and L'/L × 100% were calculated to determine the percentage position of point P on the body surface. The point on the opposite skin surface projected from point P through CINDR or CHRMSA was defined as point P'. The length of P-CINDR or P-CHRMSA and line PP' was measured, and their ratio was calculated to determine the percentage puncture depth:P-CINDR/PP' × 100% and P-CHRMSA/PP' × 100%.

### B-mode ultrasound-guided BTX-A injections

On the 3rd and 6th days after creating the rat model, 0.06 ml of 2 u/0.1 ml solution of BTX-A (Lanzhou Institute of Biological Products Co., Ltd., China) was injected into the middle of the muscle belly/CINDR/CHRMSA in MG and LG [refer to the instructions and the reference (16) for injection dosage], respectively, under B-mode ultrasound guidance (Wisonic Medical Technology Co., Ltd., China) according to the CT localization of CINDRs and CHRMSAs. The rats in each group were assigned MAS, and the BL420 biological signal acquisition device was used to measure the changes in muscle tone.

### Measurement of wet muscle weight and cross-sectional area of muscle fibers

After MAS scoring and muscle tone measurement, the rats were anesthetized by spontaneous sevoflurane inhalation and sacrificed by decapitation. The gastrocnemius muscle was dissected and weighed. Subsequently, the upper, middle, and lower parts of the INDR were stained using HE. Case Viewer software was used to measure the cross-sectional area of 500 muscle fibers within 5 fields under a microscope (the upper, lower, left, right and middle parts of the tissue section).

### Statistical analysis

All experimental data were entered into SPSS18.0 software package (IBM, US). The locations of the CINDRs and CHRMSAs were denoted in percentile measures ($\bar{x}\pm s$)%. Intergroup comparisons were performed using one-way analysis of variance, paired samples *t*-test was performed to compare the muscle tone before and after the creation of the rat model, rank sum test was used to compare the MAS among the groups. *P* < 0.05 indicates a statistically significant difference.

## Results

### Positions of CINDRs and CHRMSAs in the gastrocnemius muscles of SD rats

**Figure 1:**
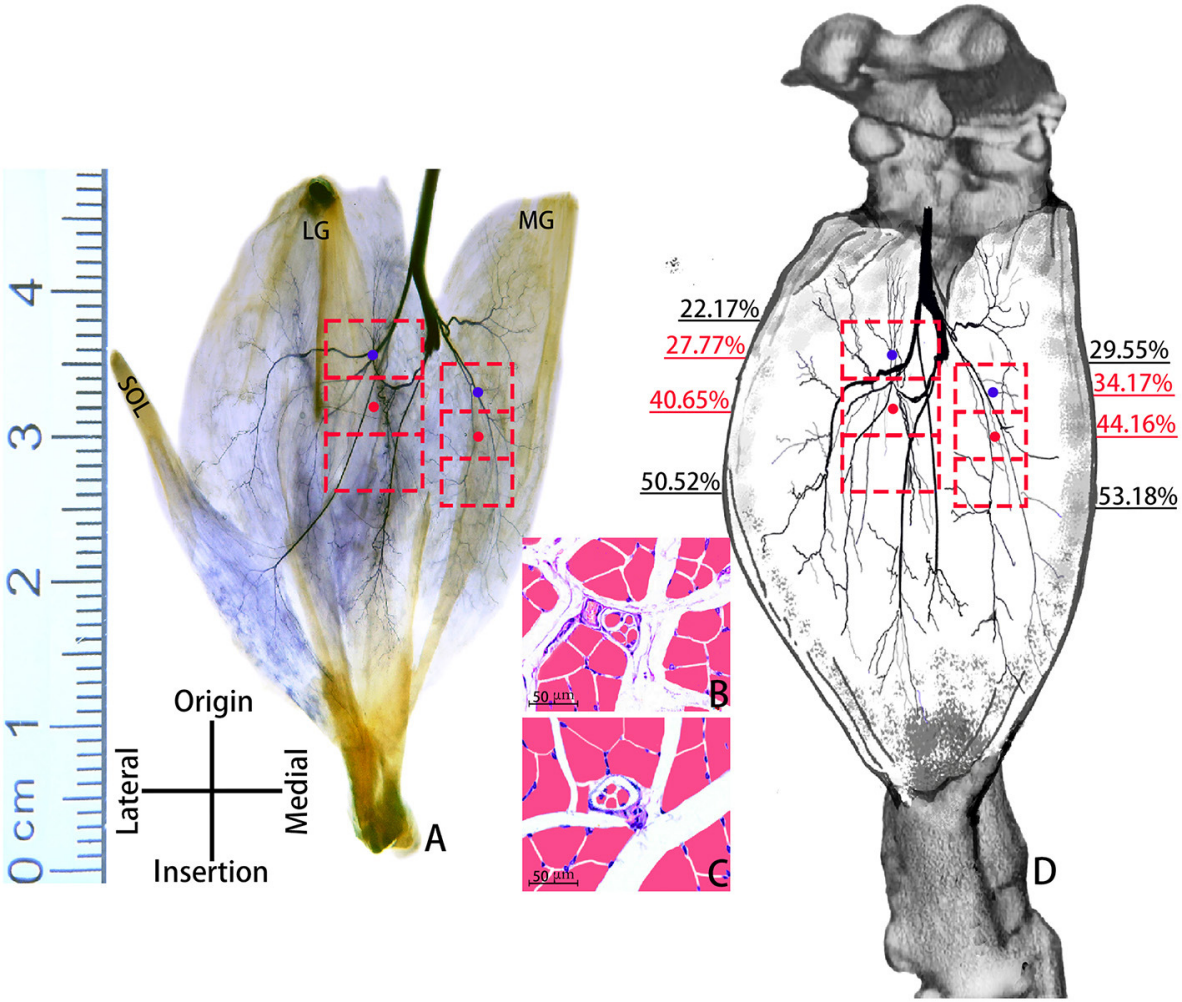
Positions of CINDRs and CHRMSAs in the gastrocnemius muscle of an SD rat. **(A)** Sihler's staining. The red boxes represent INDRs, the red dots represent CINDRs, and the blue dots represent CHRMSAs (scale bar: cm). **(B, C)** Representative muscle spindles within the upper part of the INDR in the MG and LG, respectively (scale bar: 50 μm). **(D)** Sketch of the intramuscular location of CINDRs and CHRMSAs.

Sihler's staining showed that one INDR in LG and MG was located at (22.17 ± 0.67)% to (50.52 ± 1.17)% and (29.55 ± 0.83)% to (53.18 ± 1.01)% of the muscle belly length, respectively. The corresponding CINDR was located at (40.65 ± 0.84)% and (44.16 ± 0.75)% of the muscle belly length, respectively (Figure 1). The muscle spindles were not evenly distributed across the upper, middle, and lower parts of the INDR in the LG and MG; their abundance is shown in Table 1. The upper part had the highest muscle spindle abundance among all three parts in both the LG and MG (*P* < 0.05). The differences in muscle spindle abundance between LG and MG were not statistically significant (*P* > 0.05). The CHRMSAs in LG and MG were located at (27.77 ± 0.84)% and (34.17 ± 0.79)% of the muscle belly length, respectively (Figure 1).

**Table 1:** Comparison of muscle spindle abundance across different parts of the INDR in the MG and LG.

**Parts of INDR**	**Muscle weight (g)**	**Actual number of muscle spindles**	**Predicted number of muscle spindles**	**Relative muscle spindle abundance**
LG-INDR upper part	0.83 ± 0.08	17.42 ± 1.44	18.09 ± 1.13	0.96 ± 0.02
LG-INDR middle part	0.53 ± 0.08	5.78 ± 1.38	14.16 ± 1.53	0.39 ± 0.07^Δ^
LG-INDR lower part	0.48 ± 0.06	2.59 ± 0.79	13.66 ± 1.16	0.18 ± 0.05^Δ^
MG-INDR upper part	0.57 ± 0.07	8.71 ± 1.84	14.88 ± 1.24	0.57 ± 0.09
MG-INDR middle part	0.32 ± 0.06	2.9 ± 0.59	10.82 ± 1.58	0.19 ± 0.04^▿^
MG-INDR lower part	0.23 ± 0.04	0.80 ± 0.39	9.29 ± 1.21	0.08 ± 0.03^▿^

Δ Compared with the upper part of MG-INDR;

▿ compared with the upper part of LG-INDR; *P* < 0.05.

### Body surface puncture position and puncture depth of CINDR and CHRMSA

**Figure 2:**
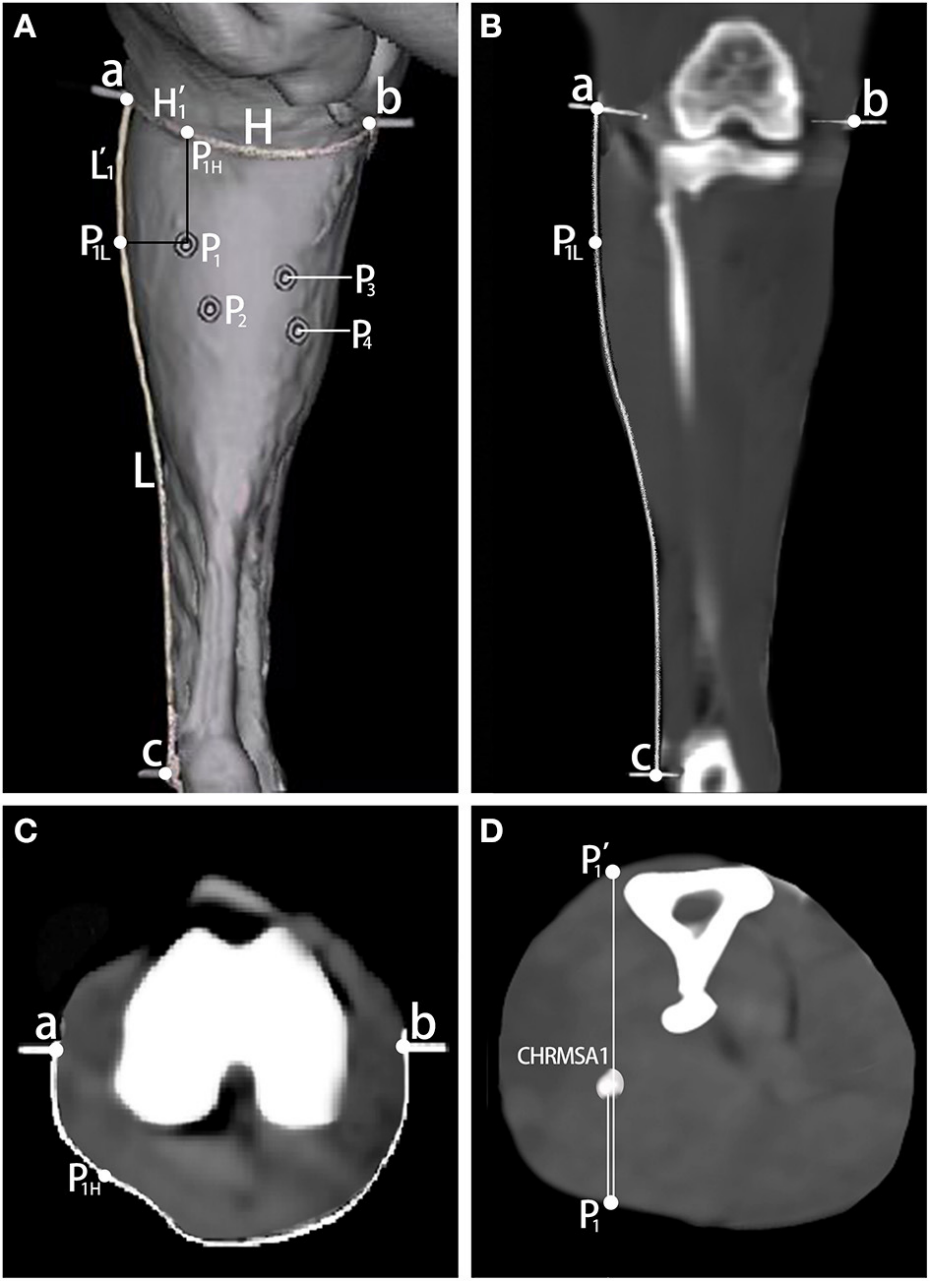
Spiral CT localization of CHRMSA in the lateral head of the gastrocnemius muscle. **(A)** Projection point of CHRMSA on the body surface and the designed reference lines. **(B)** Measurement of the lengths of the L line and the L' line on the coronal plane. **(C)** Measurement of the lengths of the H line and the H' line on the cross-sectional plane. **(D)** Measurement of the depth of CHRMSA on the cross-sectional plane.

According to the measurement, the body surface puncture points of CINDR/CHRMSA in the LG and MG were located at (20.21 ± 0.64)%/(33.33 ± 1.02)% and (28.56 ± 0.74)%/(36.91 ± 0.97)% of the L reference line, respectively, and at (28.58 ± 0.82)%/(42.85 ± 0.96)% and (68.57 ± 1.36)%/(77.14 ± 0.78)% of the H reference line, respectively. The puncture depths of CINDR/CHRMSA in the LG and MG were located at (39.68 ± 0.68)%/ (29.19 ± 1.20)% and (29.08 ± 0.65)%/(48.31 ± 0.74)% of the PP' line, respectively. Comparing the data on the left and right sides suggested that there were no statistically significant differences (*P* > 0.05). The spiral CT localization images are represented by the localization images of the CHRMSA of the LG (Figure 2).

### Evaluating success in creating rat model of muscle spasm secondary to stroke

**Figure 3:**
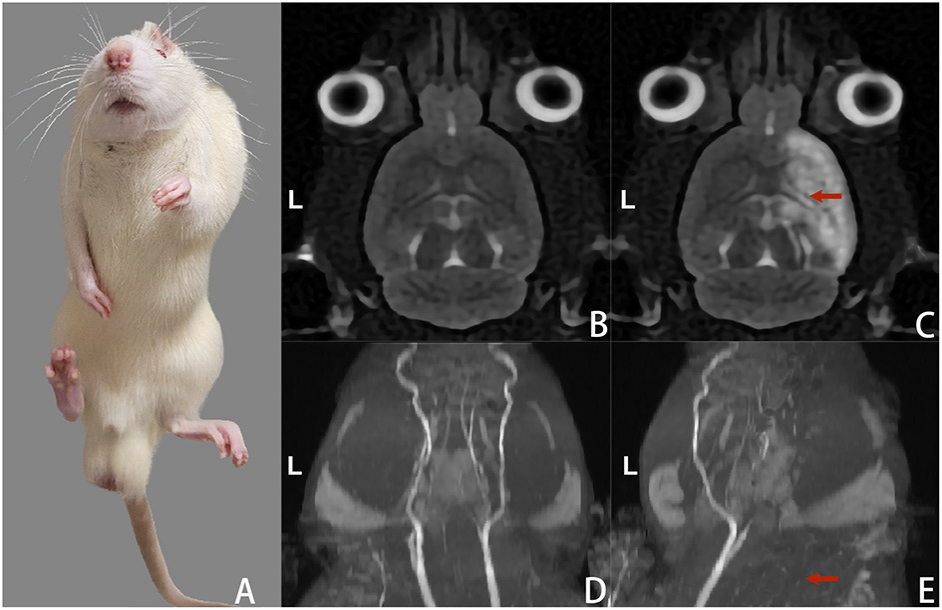
Changes in gait and head MRI scans of the rats on the 3rd day after model creation. **(A)** Spastic left limbs. **(B)** Cranial MRI scan (T_2_WI) before rat model creation. **(C)** Cranial MRI scan (T_2_WI) after rat model creation suggested high signal changes in the cerebral infarction area on the right side. **(D)** The intracranial arteries were bilaterally visualized in the MRI scan before rat model creation. **(E)** MRI scan of intracranial arteries after rat model creation suggested occlusion and non-visualization of the right-sided arteries.

On the 3rd day after creating the rat model, the rats presented with noticeable claudication, elbow flexion of the forelimb, knee flexion and plantar flexion of the hind limb, and curling of the front and rear paws on the contralateral side (Figure 3A). The MRI scans suggested patchy high signal shadow changes and occlusion in the areas supplied by the middle cerebral artery on the operated side, as well as the disappearance of the middle cerebral artery (Figures 3B–E). On the 3rd day after rat model creation before treatment, the Zea Longa scores of the rats in each group were 2.16 ± 0.75, 2.33 ± 0.82, 2.16 ± 0.75 and 2.33 ± 0.51, respectively (*P* > 0.05 for intergroup comparisons). The gastrocnemius muscle tone of the rats before model creation (4.25 ± 0.37) g was significantly lower than the average muscle tone on the 3rd day after the rat model creation before treatment (17.31 ± 0.46) g, with a statistically significant difference (*P* < 0.05).

### Evaluation of the efficacy of BTX-A injections in the treatment of muscle spasticity

**Figure 4:**
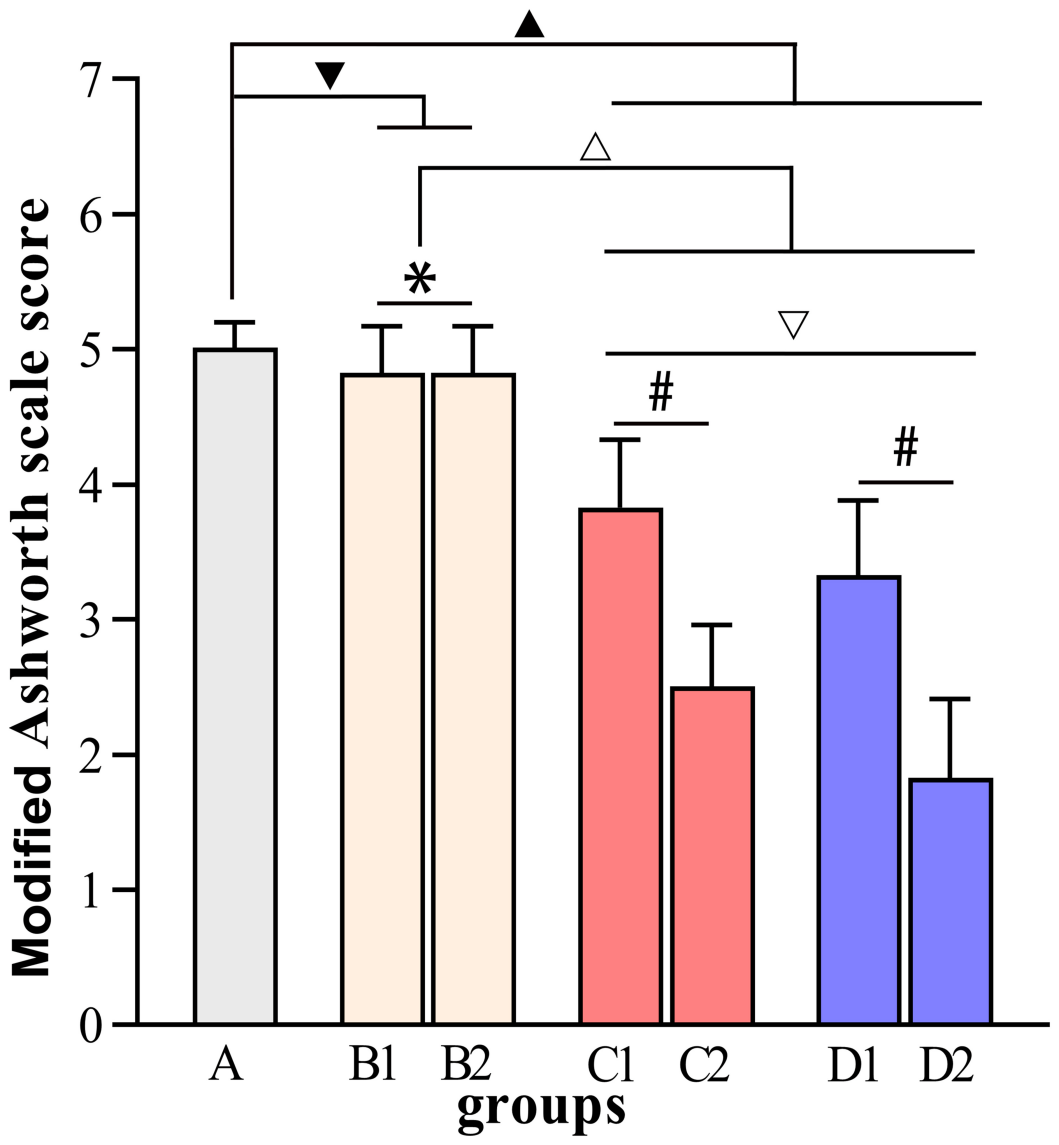
Modified Ashworth scale scores. ^▾^*P* > 0.05 in comparison with group A; ^▴^*P* < 0.05 in comparison with group A; ^Δ^*P* < 0.05 in comparison with group B; ^▿^*P* < 0.05 in comparison with group C; *P > 0.05 for the comparison between the 3rd day and 6th day after BTX-A injections; ^#^*P* < 0.05 for the comparison between the 3rd day and 6th day after BTX-A injections.

**Figure 5:**
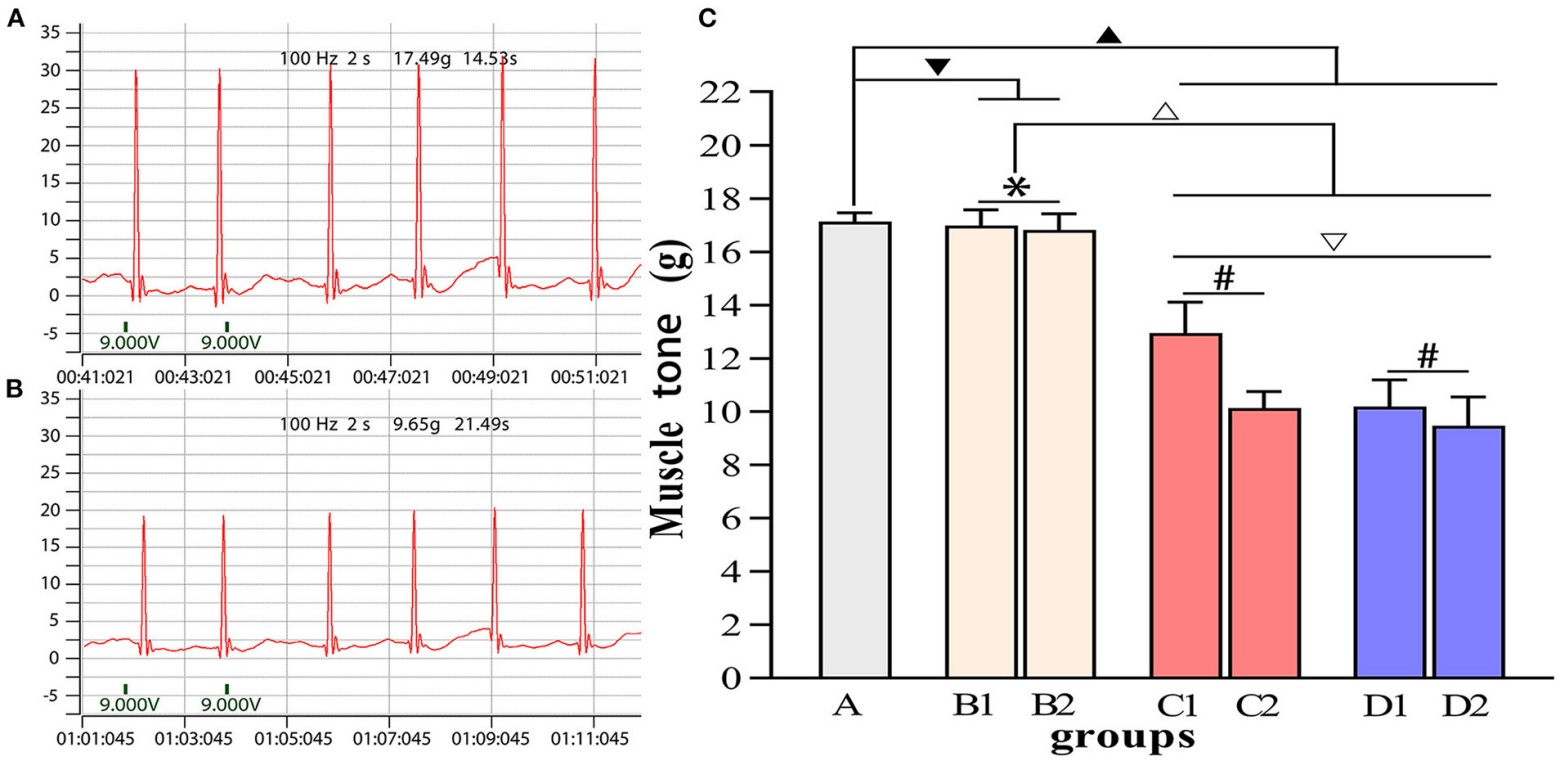
Comparisons of muscle tone in each group. **(A)** Muscle tone of group A. **(B)** Muscle tone of group D2. **(C)** Intergroup comparisons of muscle tone. ^▾^*P* > 0.05 in comparison with group A; ^▴^*P* < 0.05 in comparison with group A; ^Δ^*P* < 0.05 in comparison with group B; ^▿^*P* < 0.05 in comparison with group C; *P > 0.05 for the comparison between the 3rd day and 6th day after BTX-A injections; ^#^*P* < 0.05 for the comparison between the 3rd day and 6th day after BTX-A injections.

**Figure 6:**
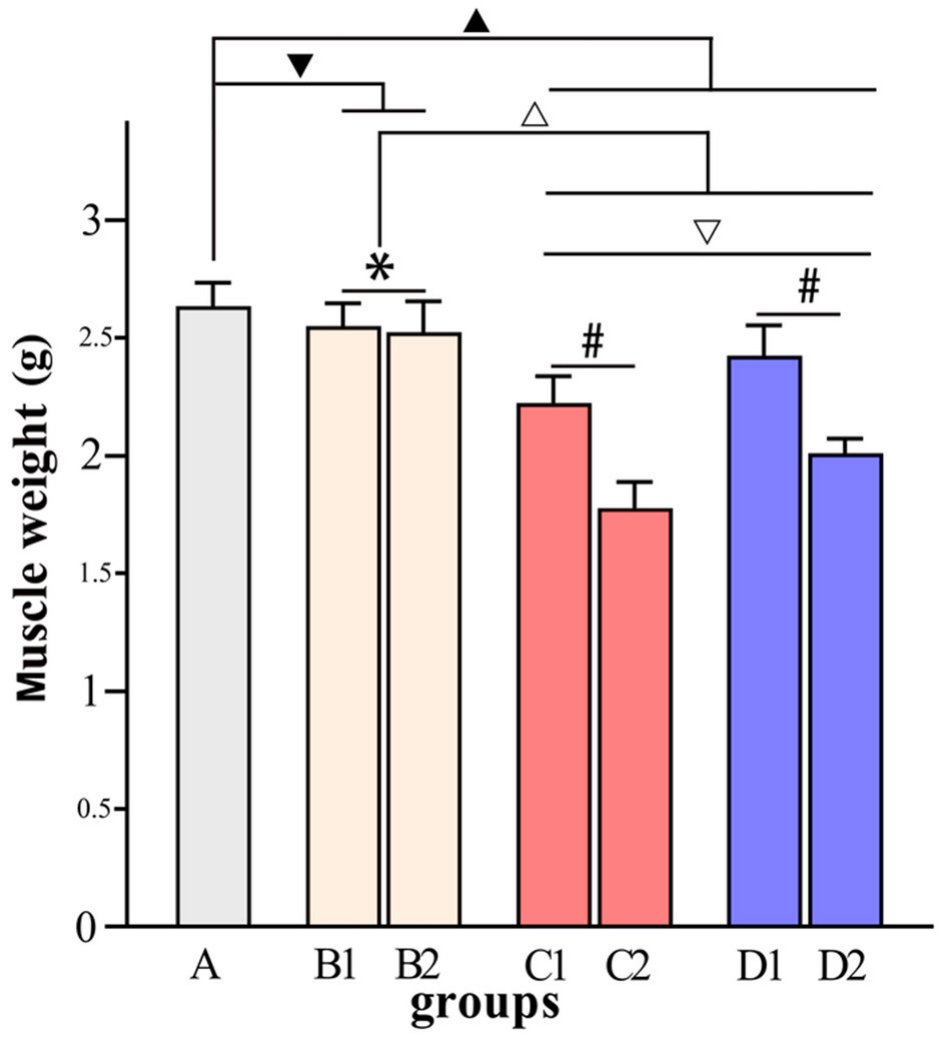
Comparisons of muscle weight in each group. ^▾^*P* > 0.05 in comparison with group A; ^▴^*P* < 0.05 in comparison with group A; ^Δ^*P* < 0.05 in comparison with group B; ^▿^*P* < 0.05 in comparison with group C; *P > 0.05 for the comparison between the 3rd day and 6th day after BTX-A injections; ^#^*P* < 0.05 for the comparison between the 3rd day and 6th day after BTX-A injections.

**Figure 7:**
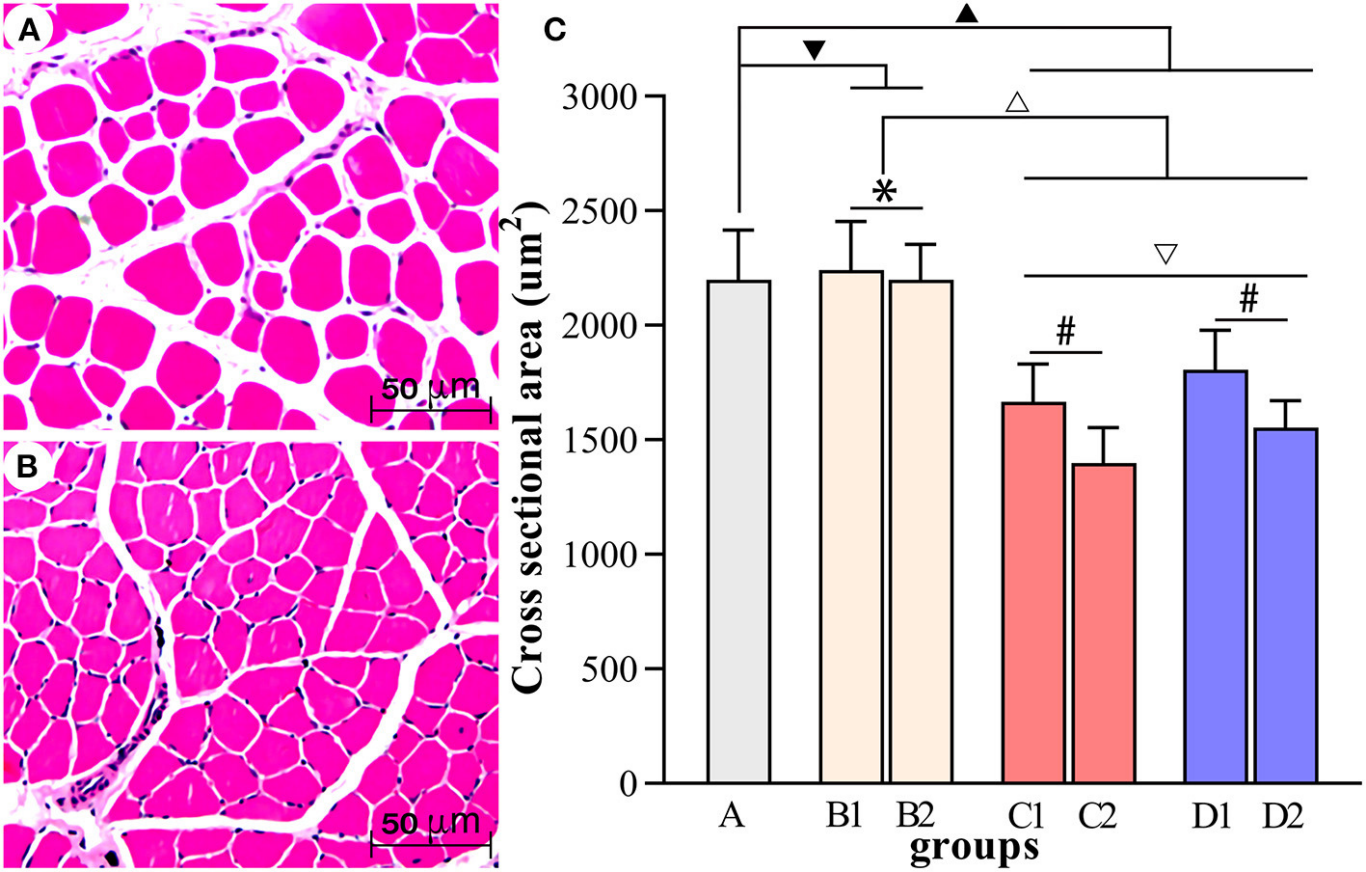
Comparisons of the cross-sectional area of muscle fibers in each group. **(A, B)** Show the HE staining of muscle fibers in group A and group D2, respectively (scale bar: 50 μm). **(C)** Comparisons of the cross-sectional area of muscle fibers in each group. ^▾^*P* > 0.05 in comparison with group A; ^▴^*P* < 0.05 in comparison with group A; ^Δ^*P* < 0.05 in comparison with group B; ^▿^*P* < 0.05 in comparison with group C; *P >0.05 for the comparison between the 3rd day and 6th day after BTX-A injections; ^#^*P* < 0.05 for the comparison between the 3rd day and 6th day after BTX-A injections.

Figures 4–7 show the changes in MAS, muscle tone, muscle weight, and cross-sectional area of muscle fibers in each group of rats after BTX-A injections. Compared with group A, group B experienced no significant changes in MAS, muscle tone, wet muscle weight or cross-sectional muscle fiber area; group C experienced the most significant decrease in wet muscle weight and cross-sectional muscle fiber area; group D experienced the most notable decrease in MAS and muscle tone (*P* < 0.05). Comparisons between the 3rd and 6th days after BTX-A injections showed that there were no significant differences in the indicators between groups B2 and B1 (*P* > 0.05), and progressive changes were observed in the indicators between groups C2 and C1 and between groups D2 and D1 with significant differences; group D had more notable intersubgroup differences than the other groups (*P* < 0.05).

## Discussion

Muscle spasticity has a high incidence rate, and the incidence of cerebral stroke is as high as 120 per 100,000 people (1). BTX-A injections into motor endplates for muscle spasticity is a practical and increasingly popular therapeutic approach; however, the locations of many motor endplate bands within human muscles are yet to be revealed. Previous theories developed by researchers suggest that INDRs are at the exact locations as motor endplate bands. Muscle spindles feature allometric growth and distribution within INDR in high abundance, thus indicating that they play an essential role in muscle spasticity. At present, botulinum toxin remains the most efficient therapy of dystonia. Its muscular mechanism of action is hinged on cholinergic blockade not only of extrafusal, but also of intrafusal muscle fibers (17). We defined the HRMSA within INDR as the optimal target of BTX-A blocking injections for muscle spasticity in the current study. The results of this study provide a new target for future BTX-A injections, help maximize the blockade of intraspindle muscle fibers, minimize the effect on extra-spindle muscle fibers (18), effectively relieve the key factors contributing to muscle spasticity, help avoid damage to the function of extra-spindle muscle fibers, and improve the quality of rehabilitation.

BTX-A is a dose-dependent neurotropic toxin, and its efficacy depends on the distance between the injection target site and the motor endplate. If the position of the BTX-A injection target site deviates from the motor endplate by 5 mm, the deviation directly reduces the antispasmodic effect by 50% (19). One unit of BTX-A can only infiltrate an area of 1.5–3 cm^2^, and 2.5–5.0 units can diffuse through an area of up to 4.5 cm^2^ (20). If multisite or high-dose injections covering large areas are administered, the therapeutic agent diffuses to the surrounding muscle tissues, thus resulting in unnecessary complications (21, 22). For example, if blepharospasm is treated with BTX-A injections into the orbicularis oculi muscle, BTX-A might diffuse to the levator palpebrae superioris and cause the sagging of eyelid muscles (23). When BTX-A injections are administered to induce temporary ptosis for cornea protection, such injections might lead to transient superior rectus underaction (24). High-dose and multisite BTX-A injections might cause superior rectus palsy when administered for the treatment of upper eyelid contractures (25). Kong et al. (26) reported that 100–500 units of BTX-A injections into the pectoralis major muscle are required to treat muscle spasticity. By contrast, our study on INDR localization suggested that (9) only 9–18 units of BTX-A are required as long as the injection target is accurately localized. If the HRMSA within INDR is accurately localized in the future, significantly lower doses of BTX-A are required to relieve the excitation of intraspindle muscles. Hence, verifying HRMSA as the optimal target of BTX-A injections for blocking muscle spasticity is essential for this study and can guide future research on the anatomical localization of BTX-A injection targets. According to the results of this experiment, the INDR of the gastrocnemius muscle is located in the upper part of the muscle belly instead of the middle part, thus suggesting that the efficacy of BTX-A injections into the middle of the muscle belly is unsatisfactory. The reason for this is the motor endplate band (at the exact location of INDR) is located in the middle of the muscle belly only when the muscle is composed of muscle fibers of equal length, and the position of the motor endplate band varies in muscles with different morphological properties (27–29). The changes in gait, Zea Longa scores, muscle tone, and MRI scans of the rats before and after model creation indicated success in creating the rat model of muscle spasticity secondary to cerebral stroke for this experiment. Compared with group A, group B experienced no significant changes in the indicators after receiving BTX-A injections, and this finding is attributed to the fact that the middle of the muscle belly is sparsely innervated and is not the injection target. Group C experienced the most significant decrease in wet muscle weight and cross-sectional area of muscle fibers, and this is attributed to the fact that BTX-A was injected into the center of the nerve-dense region and that the BTX-A injection itself can cause neurotoxic injury and resultant muscle atrophy (30). Group D experienced the most notable decrease in MAS and muscle tone, thus suggesting that BTX-A injections into the HRMSA may produce the highest efficacy. Comparisons between the 3rd and 6th days after BTX-A injections suggested no significant differences in the indicators between groups B2 and B1, as well as progressive changes in the indicators between groups C2 and C1 and between groups D2 and D1, with group D experiencing more notable intersubgroup differences. This result further proved that BTX-A injections into the middle of the muscle belly are ineffective. In addition, the efficacy of BTX-A injections into the INDR increased with an extended treatment course; however, the efficacy was still lower than the efficacy of BTX-A injections into the HRMSA.

The study on the distribution of muscle spindles in the deep cervical muscle put forward the view that the sites with more muscle spindles will receive better clinical outcomes (31), this study suggested for the first time that the efficacy of BTX-A injections into the HRMSA is significantly superior to that of conventional BTX-A injections into the middle of the muscle belly or the INDR in the treatment of muscle spasticity. We also recommend HRMSA as the optimal target of BTX-A injections for blocking muscle spasticity. However, further studies on the anatomical localization of the HRMSA within human muscles are still required. BTX-A injections into the HRMSA need to be performed in actual clinical practice on the basis of localization studies to verify its efficacy.

## Conclusion

The highest region of muscle spindle abundance should be the optimal target of botulinum toxin A injection to relieve muscle spasms.

## Data availability statement

The original contributions presented in the study are included in the article/supplementary material, further inquiries can be directed to the corresponding author.

## Ethics statement

The animal study was reviewed and approved by the Ethics Committee of the Laboratory Animal Center of Zunyi Medical University (Production No. SCXK-2021-0002; Use No. SYXK-2021-0004).

## Author contributions

SY has given substantial contributions to study conception and design. JY, YuL, LY, YiL, and SZ contributed to experiment and data acquisition, analysis, and interpretation. All authors read and approved the final version of the manuscript.
